# Out-of-hospital cardiac arrest in the home: Can area characteristics identify at-risk communities in the Republic of Ireland?

**DOI:** 10.1186/s12942-018-0126-z

**Published:** 2018-02-20

**Authors:** Siobhán Masterson, Conor Teljeur, John Cullinan, Andrew W. Murphy, Conor Deasy, Akke Vellinga

**Affiliations:** 1 School of Medicine, National University of Ireland Galway, Galway, Ireland; 2 Public Health and Primary Care, Trinity College, Dublin, Ireland; 3 School of Business and Economics, National University of Ireland Galway, Galway, Ireland; 4 National Ambulance Service, Dublin, Ireland

**Keywords:** Out-of-hospital cardiac arrest, Resuscitation, Deprivation, Residential characteristics, Spatial smoothing, Conditional autoregression

## Abstract

**Background:**

Internationally, the majority of out-of-hospital cardiac arrests where resuscitation is attempted (OHCAs) occur in private residential locations i.e. at home. The prospect of survival for this patient group is universally dismal. Understanding of the area-level factors that affect the incidence of OHCA at home may help national health planners when implementing community resuscitation training and services.

**Methods:**

We performed spatial smoothing using Bayesian conditional autoregression on case data from the Irish OHCA register. We further corrected for correlated findings using area level variables extracted and constructed for national census data.

**Results:**

We found that increasing deprivation was associated with increased case incidence. The methodology used also enabled us to identify specific areas with higher than expected case incidence.

**Conclusions:**

Our study demonstrates novel use of Bayesian conditional autoregression in quantifying area level risk of a health event with high mortality across an entire country with a diverse settlement pattern. It adds to the evidence that the likelihood of OHCA resuscitation events is associated with greater deprivation and suggests that area deprivation should be considered when planning resuscitation services. Finally, our study demonstrates the utility of Bayesian conditional autoregression as a methodological approach that could be applied in any country using registry data and area level census data.

**Electronic supplementary material:**

The online version of this article (10.1186/s12942-018-0126-z) contains supplementary material, which is available to authorized users.

## Background

Cardiac arrest occurs when the heart suddenly stops or becomes incapable of pumping blood around the body, and is the ultimate cause of all deaths [43]. Out-of-hospital cardiac arrest (OHCA) is the term ascribed to incidents that occur unexpectedly outside of an acute medical setting, and where the patient is attended by Emergency Medical Services (EMS). Survival from OHCA is almost entirely dependent on the initiation of ‘The Chain of Survival’. If the Chain of Survival is not activated within minutes of OHCA occurring, death is certain. The Chain of Survival is a sequence of resuscitation interventions, namely: early recognition of OHCA and immediate call for help to the EMS; high quality cardiopulmonary resuscitation (CPR); defibrillation within minutes of collapse; and effective advanced EMS and post-resuscitation care [34]. Timely resuscitation that is correctly performed is extremely effective. It has been shown that defibrillation within 3-5 min of collapse can result in survival as high as 50–70%. Each minute of delay however reduces the likelihood of survival by 10–12% [45]. For this reason, the ability to predict where OHCA events are most likely to occur can provide the opportunity to configure the provision of resuscitation training skills and services so that they are available in areas where they are most likely to be required.

The majority of OHCAs worldwide occur in private residential locations i.e. at home. The proportion of cases that occur at home range from 65.6% in Asia, 69.4% in Europe, 70.2% in Victoria (Australia), to 81.2% in the United States [18, 20, 42, 59]. Patient level factors have been reported to account for up to 89% of variability in OHCA outcome, including the location of collapse [8]. Whilst some of these factors cannot be affected by resuscitation services (e.g. age, gender, comorbidities), if geographic areas at higher risk of OHCA could be identified, there is potential to target these areas to improve the availability of modifiable predictors of survival at area level (e.g. bystander cardiopulmonary resuscitation (CPR) and availability of rapid defibrillation). This is particularly important when the event occurs at home, where the prospect of survival is poorer. Goh et al. [19] observed a three-fold difference in survival between home and non-residential locations (1 vs. 3% respectively). In an earlier study, Folke et al. [15] observed an even greater difference with only 3% of patients surviving at home compared with 14% in public locations. Given the proportion of OHCA that occurs at home, initiatives to narrow the gap between home and ‘not home’ survival are needed.

While the majority of OHCA events are located at home, geographic variation in OHCA incidence is consistently observed in OHCA epidemiological studies [20, 21, 38]. One aspect of geography that should be taken into account when considering variation is spatial autocorrelation, “the ubiquitous phenomenon that two close areas are often more similar than those that are far apart” [3]. Regression analyses that do not account for spatial autocorrelation are at risk of violating the assumption of independence which is generally necessary for regression analysis. The Bayesian conditional autoregression (CAR) model accounts for spatial autocorrelation in the error term, and has been shown to be particularly suited to modelling spatial phenomena strongly tied to a local context, ensuring a more realistic estimate of relative area risks [5, 28]. From a health perspective, health behaviours tend to be clustered in individuals and some of this clustering may be linked to shared neighbourhood characteristics [37, 51, 56]. This may also be the case for the occurrence of OHCA and attempted resuscitation.

In this study we aimed to estimate the underlying relative risk by small area of OHCA that occurred at home. Additionally we aimed to (1) identify underlying area-level factors that may increase the incidence of OHCA that occurs at home and (2) identify specific areas where the risk of OHCA at home is greatest in the Republic of Ireland. We also considered the influence of self-reported health, the rurality of a location, and the material deprivation of each ED on the incidence of OHCA in our analysis.

## Methods

### Setting

In the 2011 census the Republic of Ireland recorded a population of 4,588,252 [11]. The country is divided into 3409 small areas called Electoral Divisions (EDs) [13]. The average ED population was 1346 (ranging from 73 to 36,057). At the time of the 2011 national census, 62% of the population lived in urban settlements of 1500 people or more, accounting for 8% of total land mass. The remaining approximate 1.7million population were dispersed across the 65,000 square kilometres which constitute rural Ireland (Center for International Earth Science + Information Network—CIESIN—Columbia University [10].

The Irish National Ambulance Service is the sole provider of statutory Emergency Medical Services (EMS) outside of Dublin where the Dublin Fire Brigade (DFB) also provides the statutory EMS response. The Advanced Medical Priority Dispatch System (AMPDS©) is used by both NAS and DFB to prioritise calls. Emergency ambulances and rapid response vehicles are tasked to OHCA incidents and are staffed by paramedics and advanced paramedics. Intermediate care vehicles may also be dispatched as first responders or to assist in the event of OHCA and are usually staffed by emergency medical technicians. In Dublin, fire tenders are staffed primarily by fire fighter paramedics and are routinely tasked to OHCA in the greater part of the city. Irish statutory EMS staff must be licensed and registered with the Irish Pre-Hospital Emergency Care Council (PHECC) and are required to comply with PHECC Clinical Practice Guidelines in their practice (PHECC [46] 2012).

### Data

Data from the national Out-of-Hospital Cardiac Arrest Register (OHCAR) were extracted for the period 1st January 2012 to 31st December 2014 [30]. OHCAR is a register of all patients who suffer an OHCA, are attended by the EMS, and have resuscitation attempted. In Ireland, as for the majority of countries where statutory resuscitation services are provided, specific circumstances exist under which the EMS are permitted to not attempt resuscitation, including recognition of death and the presence of a ‘do not resuscitate’ order (PHECC [47] 2017). OHCAR data are extracted from ambulance Patient Care Reports (PCRs) which are completed by EMS personnel during or directly after attending the OHCA event. Dispatch and time variables for each case are obtained from NAS and DFB ambulance control centres. Case validation and registration comprehensiveness is routinely performed to ensure the quality of OHCAR data [32]. Cases where no resuscitation was attempted are not recorded in OHCAR.

Patients 18 years or older, who suffered an event of non-traumatic aetiology and were not witnessed collapsing by the EMS, were included in the study. In European Union statistics on cardiovascular diseases, death at less than 65 years is considered ‘premature’ [58]. Patients were therefore categorised into two age groups in order to check for a differing risk according to patient age i.e. less than 65 years and 65 years and older.

### Geocoding and data preparation

Private residential event location addresses were geocoded to latitude and longitude using the application ‘Health Intelligence Ireland’ (Health Intelligence Unit [22] 2015). Coordinates were then allocated to EDs using ArcGIS (Environmental Systems Research 95 Institute [ESRI] Inc., Redlands, CA). Expected rates of OHCA by small area were computed using indirect standardisation based on 2011 census population figures. Standardisation was on the basis of sex and 5 year age bands. Standardised incidence ratios were also calculated for the two age categories separately (less than 65 years (Home U65) and patients aged 65 years or older (Home 65 +)). Unsmoothed standardised incidence ratios that were greater than 1 where considered to be ‘high’ while those below 1 were considered to be ‘low’.

Three ED-level covariates were included: deprivation, urban–rural class and self-reported health. The deprivation index was based on four census indicators: unemployment, low social class, local authority housing, and car ownership [25]. The indicators were selected based on the philosophy of the Townsend index developed in the UK [62]. The four indicators are combined using principal components analysis, with the weights for the first three indicators being approximately equal, while a marginally lower weight applies to car ownership. The index is an estimate of material deprivation in an ED, and thus is a multidimensional measure of the socioeconomic status of an area. It can be expressed as a standardised score with higher positive values indicating greater deprivation, or as quintiles. While the latter results in regression coefficients that may be more interpretable, it does not capture the skewed nature of the measure, and the fact that highly deprived areas are more commonly located in city centres. We used a continuous score in the main analysis, and included a secondary analysis based on quintiles. Urban/rural classification was on the basis of a previously developed index with four levels: city; town; village; rural [61] updated in [31]. The classification combines information on population density, settlement size, land use, and proximity to other settlements. Higher rates of OHCA might be anticipated in areas where a high proportion of population report bad or very bad health. For this reason, a third variable indicating the proportion of people self-reporting bad or very bad health in each ED (Health) in the census 2011 was also calculated.

### Exploratory geographic analysis

Before performing CAR analysis, a check for spatial autocorrelation in all Home cases, and the Home U65 and Home 65 + subgroups, was carried out using the Global Moran’s *I* statistic. Global Moran’s *I* is a z-score which describes the degree of spatial concentration or dispersion for a measured variable.

### Spatial smoothing

At the ED-level, OHCA is a relatively uncommon event, and therefore incidence rates are subject to substantial variability due to small numbers. Spatial smoothing provides a method to reduce noise due to random variation. The Bayesian CAR model was used to smooth the standardised incidence rates to adjust for small numbers and to allow the model to ‘borrow strength’ from observations in neighbouring areas. The model requires data on the spatial structure of observations. The neighbourhood matrix was determined using ‘first order queen contiguity’ i.e. EDs that share a boundary were considered neighbours. Artificial links were created for EDs that were otherwise not connected (e.g. islands).

The Bayesian CAR model was fitted using Markov Chain Monte Carlo algorithms with WinBUGS. The model is based on the assumption of a Poisson model for the spatial distribution of events. The model was given a burn-in run of 10,000 iterations followed by 40,000 iterations. Convergence was tested using the Gelman–Rubin test [27].

Initial models were created for all three groups without covariates. Models were also estimated for each covariate alone, all pairings of covariates and for all three covariates. Models were estimated separately for all cases and both age group-related subcategories. Model selection was conducted using the deviance information criterion (DIC), where a lower DIC suggested a better compromise between model fit and parsimony [23]. A difference of less than 5 in model DIC is not considered sufficient to distinguish between two models [27]. Where multiple models resulted in differences of less than 5 relative to the DIC of the best fitting model, preference was given to the model with the fewest covariates. Analysis was conducted on yearly data in order to assess the sensitivity of the overall results to yearly fluctuations in the spatial distribution of events. For ease of explanation, risk ratios were calculated for covariates included in the final models.

## Results

Over a 3 year period (1st Jan 2012 to 31st December 2014), a total of 4834 OHCAR cases were eligible for inclusion in the analysis, of which 3388 cases were classified as ‘Home’ cases. Each Home case was successfully geocoded to the ED centroid level. A total of 41.6% of events occurred in patients aged less than 65 years (n = 1410). Table 1 describes the patient and ED characteristics of all Home cases.

**Table 1:** Summary characteristics of home cases (n = 3388) compared to Irish population (n = 4,588,252, Irish census 2011 [12] )

	Home cases	Irish population
Age in years [median (IQR)]	68 (55–78)	36*
Age 65 + years n (%)	1978 (58.2%)	535,393 (11.7%)
Male n (%)	2243 (66.0%)	2,272,699 (49.5%)
Number of EDs	1512 (44.3%)	3409 (100%)
*Urban*–*rural classification of EDs*		
City n (%)	395 (26.1%)	478 (14.0%)
Town n (%)	253 (16.7%)	293 (8.6%)
Village n (%)	177 (11.7%)	266 (7.8%)
Rural n (%)	687 (45.4%)	2372 (69.6%)
Average proportion of people self-reporting bad or very bad health in each ED (SD)	1.6% (1.4%)	1.4% (0.83%)
Average SAHRU deprivation score in each ED (SD)	0.4 (2.0)	0.0 (1.6)

*IQR* Interquartile range; *EDs* Electoral divisions; *SAHRU* small area health research unit

*Interquartile range not available

Unsmoothed SIRs ranged from 0 to 15.8 for all Home cases, and from 0 to 32.8 and 0 to 27.6 for the Home U65 and Home 65 + subgroups respectively. The Global Moran’s *I* statistic was calculated for the observed cases per ED. A z-score of 28.3 for all Home cases was highly significant. Similarly, z-scores of 18.7 and 26.3 for the Home U65 and Home 65 + subgroups respectively were also highly significant, confirming spatial autocorrelation in observed incidence.

Table 2 shows the results of performing Bayesian conditional autoregression on all Home cases and both age subgroups, using all possible combinations of the three covariates. While there was limited difference in the DIC for some models, the beta coefficient for deprivation (i.e. magnitude of effect of deprivation on incidence) was largely unaffected by the inclusion of other covariates. The models including only the deprivation covariate therefore were best in terms of fit and parsimony for all cases and both age subgroups.

**Table 2:** Coefficients and deviance information criteria for all models

	Beta coefficients (95% CI)					Deviance information criteria
	Deprivation	Health	City	Town	Village	
*All home cases*						
No covariates						7197.8
+ Deprivation*	0.10 (0.09, 0.12)					7117.8
+ Urban–rural			0.34 (0.16, 0.52)	0.17 (0.06, 0.28)	0.19 (0.06, 0.33)	7190.3
+ Health		0.12 (0.07, 0.16)				7187.8
+ Deprivation and urban–rural	0.10 (0.08, 0.12)		0.07 (− 0.10, 0.23)	− 0.00 (− 0.11, 0.10)	0.08 (− 0.06, 0.21)	7119.6
+ Deprivation and health	0.11 (0.08,0.13)	− 0.01 (− 0.06, 0.04)				7119.0
+ Urban–rural and health		0.10 (0.06, 0.15)	0.25 (0.07, 0.43)	0.13 (0.02, 0.24)	0.17 (0.03, 0.30)	7182.8
+ Deprivation and health and urban–rural	0.10 (0.08, 0.13)	− 0.01 (− 0.06, 0.04)	0.07 (− 0.10, 0.24)	− 0.00 (− 0.11, 0.11)	0.08 (− 0.06, 0.22)	7122.0
*Home cases* < *65*						
No covariates						4460.3
+ Deprivation*	0.14 (0.12, 0.17)					4365.8
+ Urban–rural		0.23 (0.17, 0.29)				4428.5
+ Health			0.39 (0.15, 0.62)	0.42 (0.27, 0.58)	0.39 (0.18, 0.59)	4433.8
+ Deprivation and urban–rural	0.14 (0.11, 0.17)		− 0.01 (− 0.24, 0.21)	0.19 (0.03, 0.35)	0.23 (0.02, 0.43)	4360.9
+ Deprivation and health	0.13 (0.11, 0.16)	0.05 (− 0.03, 0.13)				4365.3
+ Urban–rural and health		0.21 (0.14, 0.27)	0.20 (− 0.03, 0.42)	0.34 (0.19, 0.49)	0.33 (0.13, 0.53)	4411.4
+ Deprivation and health and urban–rural	0.13 (0.10, 0.16)	0.05 (− 0.03, 0.13)	− 0.02 (− 0.25, 0.20)	0.19 (0.03, 0.34)	0.23 (0.02, 0.43)	4361.1
*Home cases 65*+						
No covariates						5635.7
+ Deprivation*	0.07 (0.05, 0.10)					5607.0
+ Urban–rural		0.05 (0.00, 0.11)				5635.3
+ Health			0.32	− 0.01	0.07	5634.1
+ Deprivation and urban–rural	0.08 (0.05, 0.10)		0.13	− 0.14	− 0.02	5603.4
+ Deprivation and health	0.09 (0.06, 0.12)	− 0.05 (− 0.12, 0.02)				5606.6
+ Urban–rural and health		0.04 (− 0.02, 0.10)	0.29	− 0.02	0.06	5633.5
+ Deprivation and health and urban–rural	0.09 (0.06, 0.12)	− 0.06 (− 0.13, 0.01)	0.16	− 0.14	− 0.01	5603.5

*Preferred models

Table 3 shows that higher ED deprivation scores were associated with higher incidence of all Home cases and both age subgroups. For all Home Cases, an increase of one point in Deprivation was associated with an 11% increased risk of OHCA (95% CI: 9–13%). When expressed as quintiles, the risk difference between the 20% most deprived EDs and 20% least deprived EDs was 59%. The difference in deprivation was bigger for Home U65 compared to the Home 65 + category.

**Table 3:** Association of deprivation with OHCA incidence at home

	All home cases	Home U65	Home 65+
Covariate	RR (95% CI)	RR (95% CI)	RR (95% CI)
Deprivation	1.11 (1.09–1.13)	1.15 (1.13–1.18)	1.08 (1.05–1.10)

*RR* Relative rate

**Fig. 1:**
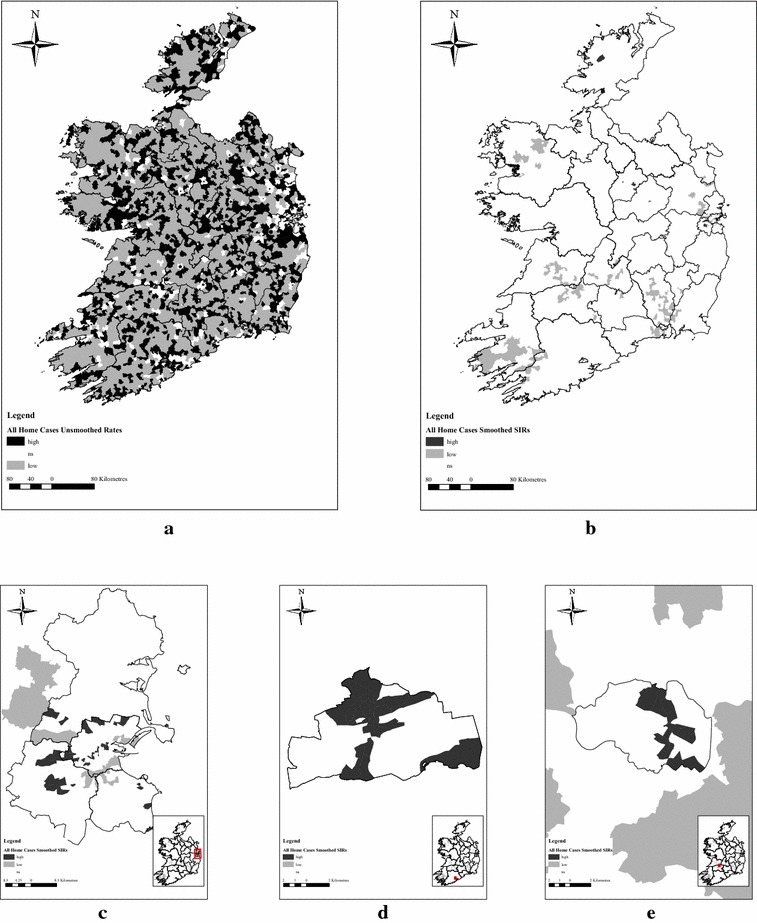
**a**–**e** Relative risk of OHCA incidence per electoral division, unsmoothed rates versus smoothed standardised incidence ratios. **a** All home cases—unsmoothed rates. **b** All home cases—smoothed standardised incidence rates. **c** All home cases—Smoothed standardised incidence rates—Dublin City and County areas. **d** All home cases—smoothed standardised incidence rates—Cork City. **e** All home cases—smoothed standardised incidence rates—Limerick City

Figure 1a displays the number of EDs with higher and lower than expected incidence ratios i.e. observed:expected incidence ratio greater than 1 or less than 1 respectively, while Fig. 1b displays the number of EDs with significantly high and low SIRs after spatial smoothing. Figure 1c–e show three cities where clusters of a higher risk of OHCA were observed after applying smoothing. Table 4 presents the number of EDs in each category of significance before and after spatial smoothing. After spatial smoothing, 100 of the 108 EDs (93%) with a higher risk of OHCA were located in Cities. This was also the case for the Home U65 subgroup (99/119; 81%) and the 65 + subgroup (55/57; 96%).

**Table 4:** Unsmoothed and smoothed standardised incidence ratios: numbers of electoral divisions categorised by significance

	All home cases		Home cases < 65		Home cases 65+	
	Unsmoothed	Smoothed	Unsmoothed	Smoothed	Unsmoothed	Smoothed
High	1122	108	717	119	936	57
Low	2227	157	2665	204	2442	82
NS	60	3144	27	3086	31	

*NS Not significant*

### Sensitivity analysis

Analysis was repeated on yearly data in order to test the sensitivity of the final All Home cases model (results available as Additional File 1: Table S1). Only five EDs were significantly high across individual years and multi-year data. Deprivation was associated with greater incidence of attempted OHCA resuscitation in annual data. However, the addition of the deprivation covariate had less effect on the DIC than when multi-year data were used. The relative risk associated with a one point increase in deprivation appeared to decrease over time, from 23% (95% CI: 17–29%) in 2012 to 10% (95% CI: 7–14%) in 2014.

## Discussion

### Key findings

Our study demonstrates the value of spatial smoothing in quantifying the area level risk of a health event with high mortality across a whole country with a diverse settlement pattern (including adjustment for area level factors). It also demonstrates novel application of Bayesian conditional autoregression to routinely collected health event registry data and area level census data. Previous authors have demonstrated the value of this methodological approach in accounting for spatial clustering in infectious diseases including dengue fever [29] and leprosy [24]. A previous Irish study used spatial smoothing to explain variation in the incidence of Amyotrophic Lateral Sclerosis (ALS), but did not attempt to provide area level explanations for the variation observed [51]. We are aware of only one other study that has analysed OHCA incidence using this methodology, in the city-state of Singapore, a considerably more uniformly populated jurisdiction than Ireland [41]. Our study addresses some of the gaps in the existing literature by showing the utility of Bayesian conditional autoregression in providing a spatial analysis (adjusted for relevant area level covariates) of a non-infectious disease event, across a whole nation with a diverse settlement pattern.

Our work adds to the evidence that the likelihood of OHCA resuscitation events—specifically in the home—is associated with greater deprivation and suggests that area deprivation should be considered when planning resuscitation services. Other covariates were not associated with OHCA incidence. By correcting for correlated findings between areas and using a methodology that accounts for spatial variation, our study considerably strengthens the evidence that deprivation is associated with a higher incidence of OHCA.

### Choice of area level covariates

Our choice of area level covariates was based on availability of data at ED level, robustness and relevance to the Irish population. In a study using a similar methodological approach, Ong et al. [41] investigated spatial variation in OHCA incidence in the city-state of Singapore. Ong et al. used a number of single measure variables including education, working status, and household size. They found no association between area level measures and OHCA incidence and suspected that small sample size may have influence the lack of association found. We opted to use a robust composite deprivation variable in order to avoid the risk of multicollinearity between single measure variables and to ensure sufficient area level data availability.

Previous studies of OHCA incidence have measured rurality as a direct function of population density and have observed reducing OHCA incidence with decreasing population density [33, 50, 57]. We chose to use a composite measure that was specifically designed to additionally account for the geographical qualities of an area, including population density, land use, proximity to an urban area and settlement size. In view of the fact that non-traumatic OHCA almost invariably occurs as the result of an underlying disease, we believed that inclusion of an area-level health measure was essential in our analysis. As area level statistics on morbidity were not available, self-reported health status was considered the most appropriate proxy measure for area level health status.

In the United States, higher OHCA incidence has been observed in neighbourhoods with a higher proportion of black race [16]. Associations between incidence and other ethnicities have not been observed. Only 1.4% of the Irish population was reported as black in the 2011 census, meaning area level analysis of race and OHCA in the Irish context would be based on extremely sparse data and unlikely to be robust.

### Association of deprivation and OHCA incidence

Our findings support those of Reinier et al. [48, 49] that there is an association between increased deprivation and increased OHCA incidence. While previous studies have found deprivation to be predictive of self-reported health [63], in our study population, deprivation appeared to be the more robust covariate, across all cases and both age subgroups. By using data from an OHCA register with national coverage, and accounting for spatial variation, our study lends support to the understanding that deprivation is associated with the incidence of OHCA incidents where resuscitation is attempted, regardless of age or geographical location.

It is important to keep in mind that deprivation is a relative concept to the country and/or circumstances in which it is measured. While individual components of a deprivation index may be stable across urban and rural areas, it cannot be assumed that an index will behave in as stable a manner as its constituent components [17]. Additionally, deprivation measures that are centred on typical urban values are less likely to correctly identify deprivation in a rural setting [2, 4]. The index used here was developed to be applicable nationally, with only one indicator included (car ownership) that might be considered to bias towards urban areas. However, given the lower weight applied to that indicator, the influence it has on the deprivation score is moderated. The covariate coefficient for deprivation in the model is largely unchanged by the addition of the urban–rural index as a covariate, supporting the view that the index is not overly biased towards urban or rural areas. Area level deprivation is not discrete. It is influenced by the deprivation level of surrounding areas, highlighting the importance of using analytical methods that account for spatial autocorrelation [7]. Even when overall affluence increases, the relative association between deprivation and health inequalities remains [26].

Considering that the most common cause for non-traumatic adult OHCA is cardiovascular disease (CVD), and greater deprivation is associated with increased incidence of CVD, an association between OHCA and deprivation is consistent with expectations [1, 35]. As aging exerts an independent effect on CVD prevalence, the smaller influence of deprivation in the older age group can also be expected [40]. In considering the impact of area level deprivation, it is important to remember that deprivation affects morbidity and mortality, which in turn influence and/or limit the health choices that people can make [60]. It is therefore ultimately individual factors, such as pre-existing morbidity and health behaviours that account for the greater proportion of incidence risk. For example, for myocardial infarction—the most common CVD precursor to OHCA—nine individual-level modifiable risk factors were found to account for 90% of the Population attributable risk (PAR) in men and 94% of PAR in women [64].

### Spatial smoothing changes the view of the geography of OHCA resuscitation

Spatial smoothing greatly reduced the number of EDs where incidence was higher or lower than expected, and enabled the identification of specific areas with significantly higher incidence. Areas with significantly higher incidence were primarily located in EDs that were classified as City. This was unexpected as 57.1% of Home Cases occur in EDs classified as either Rural or Village. According to the Irish census in 2011, only 36% of the general population were resident in Rural or Village EDs [12]. Efforts to improve community first response tend to be focussed on more remote and rural areas. Our results suggest that certain city communities with greater deprivation should also be targeted.

### Why do patients who collapse at home have poorer survival than those who collapse in a public place?

Various reasons for the difference in survival between collapsing at home have been suggested. Daya et al. [14] found that in an American population, collapse in a public location was an independent predictor of OHCA survival, even after adjusting for known predictors of OHCA survival. In Ireland we have previously reported a strong adjusted association between survival and collapse in a public place [33]. In contrast, Nakanishi et al. [36] found that the influence of home as the incident location in Japan was eliminated when adjusted for ambulance call-response interval, performance of bystander cardiopulmonary resuscitation (CPR) and initial cardiac rhythm. An unmeasured but possible explanation for this difference may also be the higher prevalence of coronary heart disease in Western countries [52].

### How these findings impact service provision

When cardiac arrest occurs, the heart becomes incapable of circulating blood around the body. In the absence of good quality cardiopulmonary resuscitation (CPR), the brain will become starved of oxygen within 5 min and cell death will begin to occur [44]. The chances of patient survival therefore are largely determined by the actions of bystanders within the first few minutes of collapse [39]. Home is the most common location of OHCA, and unless someone in the home is able to perform effective bystander CPR, the prospects of survival are severely limited. Previous studies have found an association between low income neighbourhoods and a reduced likelihood of bystander CPR being performed [53, 54]. At present, we do not know if the level of CPR training and knowledge of OHCA recognition in the general population in Ireland follows a socioeconomic gradient. Considering the association between OHCA incidence and deprivation suggested in this study and previous studies, an understanding of the association between CPR knowledge and deprivation is an important area for further research.

In Ireland—as in other countries—EMS call-response intervals increase with increasing rurality of the event [33]. Communities in more rural locations have responded to this problem with the establishment of rapid response schemes and involvement of general practitioners in the emergency response [9]. Our study shows however, that the effect of urban–rural status on OHCA incidence reduces after adjusting for deprivation. We have also identified specific areas with significantly higher incidence—the majority of which are located in cities. Zijlstra et al. [65] have shown the value of lay rescuers responding to OHCA events in densely populated residential areas. Blom et al. [6] have shown incremental improvement in OHCA survival with the introduction of police CPR training and the equipping of police vehicles with AEDs in the city of Amsterdam. It can be suggested that similar interventions could be trialled in the deprived City EDs identified in our study as being at higher risk of OHCA.

### Limitations

There are a number of limitations to our study. Firstly, OHCAR includes only OHCA where resuscitation was attempted and does not reflect the incidence of all Irish OHCA. Secondly, it was not possible in this study to consider the incidence of OHCA resuscitation which occurred at a location other than home as we could not determine a robust reference population for these cases. It is possible that areas with a low incidence of at home OHCA may have a high incidence of ‘not home’ OHCAs, in which case different policy approaches may be necessary to ensure immediate, effective resuscitation is available for cases that occur in more public locations. Thirdly, our sensitivity analysis showed differences in the EDs that were identified on a year-to-year basis. The numbers of cases in the annual analyses were small and therefore not as robust as the multi-year analysis. Additionally, OHCA was a relatively rare event and sensitivity analysis shows that areas may change on a yearly basis, which may have resulted in the improved fit of the multi-year analysis. Fourthly, deprivation is right skewed and a quintile can encompass areas with a very broad range of scores. To assume that the effect of deprivation is equal across all areas within a quintile may be unreasonable, which is why we used the score itself. Scores are used for small area studies where relative rather than absolute difference is of particular interest [7, 55]. The trade-off for using the score is that a one point change is difficult to interpret and cannot be readily considered in terms of quintiles. Finally, 1897 (56%) of the total 3409 EDs had zero Home cases—which may also have affected the robustness of our results.

## Conclusions

In conclusion, we have shown that the likelihood of OHCA where resuscitation is attempted is likely influenced by deprivation, and have demonstrated a methodology that allows the identification of specific areas of high risk by correcting for correlated findings. Additionally, our study provides the opportunity to highlight that OHCA is not an event that happens to ‘others’ but rather an event that is most likely to occur at home, often in the presence of family or friends. While public policy should be targeted to at-risk communities, the universally greater risk of collapse at home must be communicated, regardless of geography.

## Additional file

**Additional file 1: Table S1.** Deviance information criteria, beta coefficients and relative rate for All Home cases categorised by year.
