# Supplementary material for: Out-of-hospital cardiac arrest in the home: Can area characteristics identify at-risk communities in the Republic of Ireland?
Source: Int J Health Geogr. 2018 Feb 20;17:6. doi: 10.1186/s12942-018-0126-z (PMC5819205; doi:10.1186/s12942-018-0126-z)
Supplement: Supplementary file 1 — Additional file 1: Table S1. Deviance information criteria, beta coefficients and relative rate for All Home cases categorised by year. [file 12942_2018_126_MOESM1_ESM.docx]

Additional File 1: Table S1 Deviance information criteria, beta coefficients and relative rate for annual All Home cases

|  |  | **Deviance Information Criteria** | **Beta Coefficient (95% Confidence Intervals)** | **Relative Rate (95% Confidence Intervals)** |
| --- | --- | --- | --- | --- |
| **All Home cases 2012** | |  |  |  |
|  | No covariates | 4272.4 |  |  |
|  | + Deprivation | 4244.5 | 0.21 (1.16-0.25) | 1.23 (1.17-1.28) |
|  |  |  |  |  |
| **All Home cases 2013** | |  |  |  |
|  | No covariates | 4555.7 |  |  |
|  | + Deprivation | 4530.2 | 0.14 (0.10-0.18) | 1.15 (1.10-1.20) |
|  |  |  |  |  |
| **All Home cases 2014** | |  |  |  |
|  | No covariates | 4156.1 |  |  |
|  | + Deprivation | 4124.9 | 1.10 (0.07-0.13) | 1.10 (1.07-1.14) |
|  |  |  |  |  |
